# Immune checkpoint inhibitor increased mortality in lung cancer patients with *Pneumocystis jirovecii* pneumonia: a comparative retrospective cohort study

**DOI:** 10.3389/fonc.2024.1398357

**Published:** 2024-07-05

**Authors:** Bo Fan, Xiaoyan Sun, Weijie Han, Yimin Zou, Fei Chen, Fen Lan, Wen Li, Yanxiong Mao

**Affiliations:** ^1^ Department of Respiratory and Critical Care Medicine, First People’s Hospital of Jiashan, Jiashan, Zhejiang, China; ^2^ Department of Gynaecology and Obstetrics, Women’s Hospital School of Medicine Zhejiang University, Hangzhou, Zhejiang, China; ^3^ Department of Emergency, People’s Hospital of Haiyan, Haiyan, Zhejiang, China; ^4^ Key Laboratory of Respiratory Disease of Zhejiang Province, Department of Respiratory and Critical Care Medicine, Second Affiliated Hospital of Zhejiang University School of Medicine, Hangzhou, Zhejiang, China

**Keywords:** immune checkpoint inhibitor, lung carcinoma, Pneumocystis jirovecii pneumonia, mortality, metagenomics next-generation sequencing

## Abstract

**Introduction:**

*Pneumocystis jirovecii* pneumonia (PJP) is a life-threatening infection in immunocompromised individuals. Immune checkpoint inhibitor (ICI) has brought significant survival benefit in lung cancer patients. Although the few studies showed there was high mortality in PJP patients with ICI use, these studies had no comparative control groups.

**Methods:**

A retrospective study was conducted to compare the mortality in PJP patients with lung cancer between those treated with ICI and a concurrent control group treated without ICI.

**Results:**

A total number of 20 non-human immunodeficiency virus (HIV) patients with confirmed PJP and co-existing lung cancer were included in the current study, and classified into ICI group (n=9) and non-ICI group (n=11).There was a clear trend to a shorter onset of PJP in ICI group than non-ICI group (118.9 ± 60.9 vs 253.0 ± 185.1 days), although without statistical significance (p=0.053). Bronchoscopic alveolar lavage fluid were collected from all patients and used to identify *Pneumocystis jirovecii*. In both groups, metagenomics next-generation sequencing (mNGS) were the most used diagnostic techniques. Within 28 days after the onset of PJP, mortality was significantly higher in the ICI group than non-ICI group (33.3% vs 0, p=0.042)

**Conclusion:**

Lung cancer patients with ICI use had a higher mortality rate after PJP infection than patients without ICI use. Prospective studies with larger sample size and a multi-center design are warranted to further verify the present results.

## Introduction

*Pneumocystis jirovecii* (PJ) is an opportunistic pathogen that is responsible for life-threatening manifestations of *Pneumocystis jirovecii* pneumonia (PJP) in immunocompromised individuals (1). PJP remains the most prevalent opportunistic infection in patients infected with the human immunodeficiency virus (HIV) (2). In recent years, with increasing use of corticosteroids and/or immunosuppressive agents, chemotherapy and radiotherapy for malignancies, and advancement of organ transplantation, PJP has been increasingly reported in non-HIV patients as well (3–5). The prognosis of non-HIV-infected PJP patients tends to be worse, and the reported mortality of PJP in immunocompromised non-HIV patients ranges from 48% to 67% (6). Recently, because wide application of molecular diagnostic techniques has made timely diagnosis and prompt treatment a reality, the mortality of PJP have been greatly reduced (7). But PJP is still a health threat to immunocompromised individuals.

Lung cancer is a malignancy with high prevalence and mortality worldwide. PJP could occur in lung cancer patients (8). A retrospective study in France showed that 3% of non-HIV patients with PJP had lung cancer (9). Another study in Japan showed that in non-HIV solid tumor patients with PJP, lung cancer was the most common underlying tumor, which accounted for 30% of PJP cases (10). Like other non-HIV-infected PJP patients, lung cancer patients with PJP had poor prognosis. A retrospective analysis by Lee et al. revealed that lung cancer patients with PJP had an all-cause mortality rate of 61.6% during 3-month PJP treatment (11). So the high mortality of PJP in lung cancer patients warrant attention from physicians.

In recent years, immune checkpoint inhibitor (ICI) has revolutionized the treatment of lung cancer and brought significant survival benefit (12). Their use has been widely recommended in lung cancer patients by major guidelines. Since the introduction of ICI into clinical practice, concerns have emerged regarding their potential to cause infection. Now increasing evidence show that ICI use might not increase in risk of infection, but it might increase risk of infection in patients developing immune-related adverse events (irAE) and treated with additional immunosuppressive such as corticosteroids (13–15). In melanoma patients with ICI treatment, bacteria were the most common pathogen of serious infection, followed by fungus, virus and parasite (13).The study by Malek et al. showed that in lung cancer patients treated with ICI, pneumonia was the most common infection encountered, and bacteria were the dominant type of pathogens, followed by virus and fungus (14).

A meta-analysis, which included a total of 21,451 cancer patients from 36 studies, showed that ICI were associated with a similar risk of infections versus non-ICI treatments (16). So these findings have greatly relieved the concern about ICI’s detrimental effect on infection. But the concerns persist in patients with use of corticosteroids, who had increased risk of infection.

So far PJP has been reported in patients with ICI use, but the clinical features and prognosis of PJP with ICI use remains mostly unknown. There were only over a dozen PJP cases associated with ICI reported in literature. In an analysis base on the Food and Drug AdministrationAdverse Event Reporting System (FAERS) database of Food and Drug Administration (FDA), researchers identified 677 reports of PJP associated with ICI, in which 300 (44.3%) PJP cases with fatal outcome (8). They also found that male gender and age >65 years were predominant in PJP cases associated with across all ICI. Although the few studies showed there was high mortality in PJP patients with ICI use, these studies had no comparative control groups. To better evaluate the mortality risk of ICI in patients with lung cancer, we compared the mortality in PJP patients with lung cancer between those treated with ICI and a concurrent control group treated without ICI.

## Methods

### Ethical approval

This was a retrospective study of patients conducted in an academic teaching tertiary hospital (The Second Hospital of Zhejiang University School of Medicine, China). The ethical approval was sought and granted by Ethics Committee of Second Affiliated Hospital of Zhejiang University School of Medicine (Approval Number: 2023–0847). As the non-interventional retrospective study was determined to be no greater than minimal risk, the Ethics Committee of Second Affiliated Hospital of Zhejiang University School of Medicine issued a waiver of informed consent. Patient data privacy and confidentiality were maintained as this study was conducted in compliance with the ethical standards of the Declaration of Helsinki.

### Patient selection

All patients admitted to the study hospital with a discharge diagnosis of PJP between January 2017 and February 2022 were retrieved from the Electrical Medical Records System (EMRS). Patients with prior HIV infection were excluded from the study. Records were further reviewed by two pulmonologists (FL and YMZ) to confirm the diagnosis of PJP. When the opinions differed, a third pulmonologist (WL) was involved in decision. The diagnosis of PJP were made according to clinical manifestations, imaging examinations, and microbiological test results as described before (17). The criteria were as follows: (1) compatible clinical symptoms including fever, cough, sputum, and dyspnea; (2) radiological findings compatible with PJP such as uni- or bilateral ground-glass opacity or patchy consolidation; and (3) microbiologic finding including conventional or immunofluorescence staining, and molecular diagnosis by polymerase chain reaction (PCR) or metagenomics next-generation sequencing (mNGS) via respiratory specimens (sputum specimens or bronchoalveolar lavage fluid) and blood samples.

### Data collection

Demographic data, lab test results on admission, disease comorbidities, and pharmacotherapy were collected from EMRS. ICI included programmed cell death protein 1 (PD-1) agents and programmed cell death receptor ligand-1 (PD-L1) agents. The survival status of patients was assessed by medical record review and phone interview in late August 2023.

### Data analysis

The results were analyzed using International Business Machines Corporation (IBM) SPSS Statistics 20. Continuous data was presented as the mean with standard deviation (SD) or median with interquartile range (IQR), depending on the distribution of data. Variables were compared using the unpaired Student’s t-test, Welch t-test or the Wilcoxon rank sum test with continuity correction, depending on data normality and homogeneity of variance. Categorical data were presented as absolute value and percentage, and analyzed using Chi-square test or Fisher’s exact test according to test assumptions. Statistical significance was set at p< 0.05.

## Results

**Figure 1:**
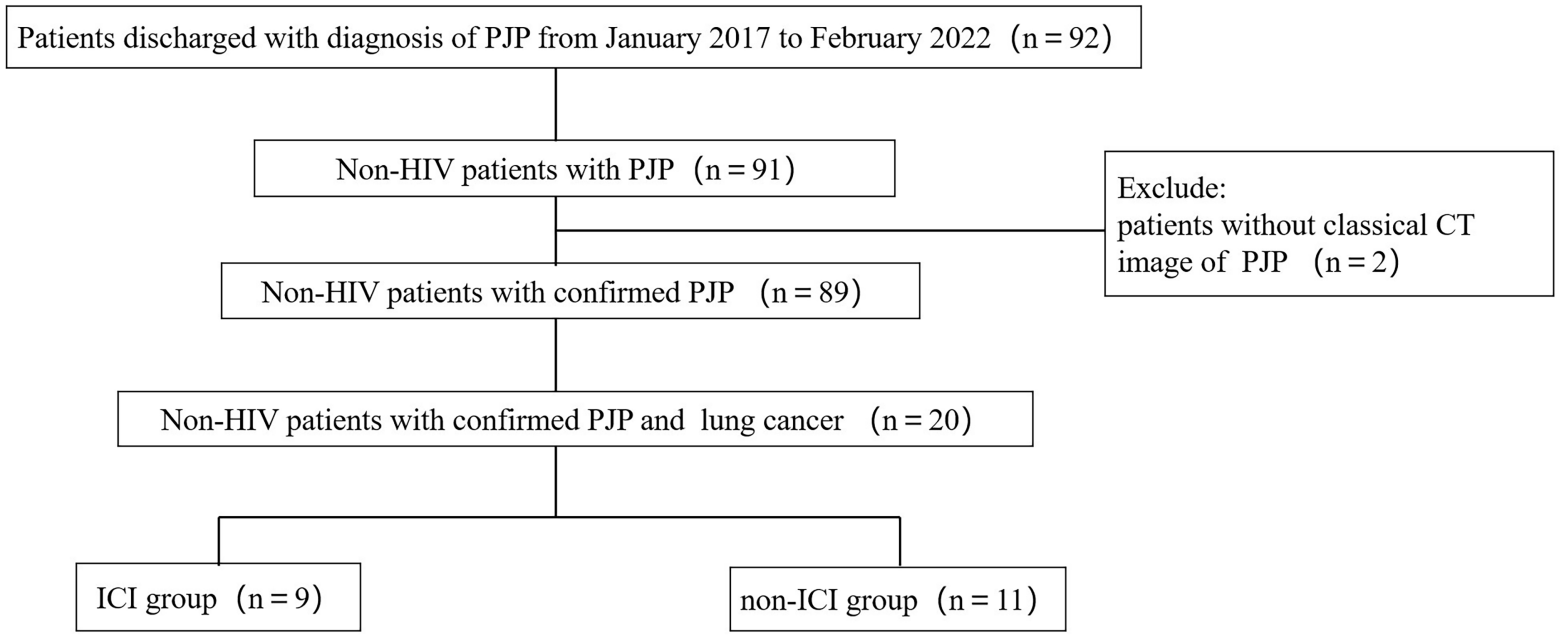
Flow chart of study population. PJP, *Pneumocystis jiroveci* pneumonia; HIV, human immunodeficiency virus; ICI, immune checkpoint inhibitor.

A total of 92 patients discharged with diagnosis of PJP between June 2017 and February 2022 were extracted from the EMRS. After screening, a total number of 20 non-HIV patients with confirmed PJP and co-existing lung cancer were included for further analysis (**Figure 1**). Of these 20 patients, there were 9 patients who had a history of ICI use (ICI group) and 11 patients who had no history of ICI use (non-ICI group).

### Baseline characteristics

Baseline demographics, comorbidities, and lung function test results were similar between two groups, except for body mass index (BMI) (**Table 1**). The ICI group had an average age of 69.11 ± 4.99 which was similar to non-ICI group (average age of 66.27 ± 6.20). The majority of patients in both groups were males (non-ICI group vs ICI group: 81.8% vs 100%). BMI were within the normal adult range in both groups, although ICI group had significantly higher BMI than non-ICI group (23.53 ± 2.45 vs 20.75 ± 2.17, p=0.015). The most common comorbidities in both groups were chronic obstructive pulmonary disease (COPD) (non-ICI group vs ICI group: 36.4% vs 44.4%) and hypertension (non-ICI group vs ICI group: 36.4% vs 44.4%). There were 2 patients (22.2%) with renal insufficiency in the ICI group, and none in the non-ICI group.

**Table 1:** Baseline demographics, comorbidities and lung function test results.

Variables	Non-ICI group (n=11)	ICI group (n=9)	p
Age	66.27 (6.20)	69.11 (4.99)	0.282
Male	9 (81.8%)	9 (100%)	0.167
BMI	20.75 (2.17)	23.53 (2.45)	**0.015**
Smoking history			0.638
Ever	4 (36.4%)	2 (22.2%)	
Current	5 (45.4%)	6 (66.6%)	
Never	2 (18.2%)	1 (11.1%)	
**Pack-years**	40 (30.00, 40.00)	40 (30.00, 47.50)	0.648
Comorbidities			
COPD	4 (36.4%)	4 (44.4%)	0.888
Asthma	0	0	**—**
ILD	0	0	**—**
Hypertension	4 (36.4%)	4 (44.4%)	0.731
Diabetes mellitus	0	0	**—**
Renal insufficiency	0	2 (22.2%)	0.169
Lung function test#			
FEV1	2.09 (0.46)	1.94 (0.76)	**—**
FEV1% predicted	85.05 (16.08)	70.08 (22.79)	**—**
FVC	2.75 (0.52)	2.91 (0.80)	**—**
FVC % predicted	90.42 (17.85)	81.35 (17.99)	**—**
DLCO % predicted	5.17 (0.44)	4.56 (2.03)	**—**
No spirometry performed	4 (36.4%)	6 (66.6%)	**—**

All data are presented as No. (%), median (interquartile range), or mean (standard deviation).

BMI, body mass; COPD, chronic obstructive pulmonary disease; ILD, interstital lung disease; FEV1, forced expiratory volume in one second; FVC, forced vital capacity; DLCO, carbon monoxide diffusing capacity.

^#^The statistical analysis was not performed due to very small sample size. "—", means that no statistical analysis can be performed between two groups due to very small sample size or no comparison.

### History of lung cancer and ICI use

The cancer subtypes and stage were similar between both groups. The percentage of patients receiving chemotherapy and surgery in both groups were similar as well (**Table 2**). The ICI group were less likely to receive chest radiotherapy than non-ICI group with borderline significance (44.4% vs 90.1%, p=0.05). Five patients (45.4%) in non-ICI group used corticosteroids prior to onset of PJP due to radiotherapy associated adverse events. Three patients (33.3%) in ICI group used prior corticosteroids. Of those 3 patients, two patients used corticosteroids due to interstitial pneumonia, and one patient used corticosteroids due to acute exacerbation of COPD. The ICI used in ICI group were as follows: tislelizumab (33.3%), pembrolizumab (22.2%), camrelizumab (22.2%) and sintilimab (22.2%).

**Table 2:** History of lung cancer and ICI use.

Variables	Non-ICI group (n=11)	ICI group (n=9)	p
Histology			0.465
Adenocarcinoma	5 (45.4%)	2 (22.2%)	
Squamous cell carcinoma	4 (36.4%)	5 (55.5%)	
Small cell carcinoma	2 (18.2%)	1 (11.1%)	
Others	0	1 (11.1%)	
Cancer Stage			0.463
II	2 (18.2%)	0 (0%)	
III	6 (54.5%)	4 (44.4%)	
IV	3 (27.3%)	5 (55.5%)	
Prior cancer treatment			
Thoracic surgery	4 (36.4%)	3 (33.3%)	1.00
Thoracic radiotherapy	10 (90.1%)	4 (44.4%)	0.050
Chemotherapy	10 (90.1%)	9 (100%)	1.00
Corticosteroids use prior to onset of PJP			
Use of corticosteroids	5 (45.4%)	3 (33.3%)	0.67
Daily dose of corticosteroids	28.80 (20.26)	17.67 (19.50)	0.475
Cause of corticosteroids use			
Radiotherapy associated adverse events	5 (45.4%)	—	
Interstitial pneumonia	—	2 (22.2%)	
AECOPD	—	1 (11.1%)	
ICI			
Pembrolizumab	**—**	2 (22.2%)	**—**
Camrelizumab	**—**	2 (22.2%)	**—**
Tislelizumab	**—**	3 (33.3%)	**—**
Sintilimab	**—**	2 (22.2%)	**—**

All data are presented as No. (%), median (interquartile range), or mean (standard deviation).

ICI, immune checkpoint inhibitors; PJP, P. jirovecii pneumonia; AECOPD, acute exacerbation of chronic obstructive pulmonary disease.

"—", means that no statistical analysis can be performed between two groups due to very small sample size or no comparison.

### PJP characteristics and treatment

There was a clear trend to a shorter onset of PJP in ICI group, although without statistical significance (ICI group vs non-ICI group: 118.9 ± 60.9 vs 253.0 ± 185.1 days, p=0.053) (**Table 3**). The CURB65 score was not different between two groups, which indicated that the severity of PJP between two groups was similar. Bronchoscopic alveolar lavage fluid (BALF) were collected from all patients and were the specimens from which PJ were identified. In both groups, mNGS were the most used diagnostic techniques (ICI group vs non-ICI group: 72.7% vs 66.6%). The cellular immunity profile was similar between two groups. Corticosteroids treatment after diagnosis of PJP were similar. Patients in both groups were treated with trimethoprim/sulfamethoxazole (TMP-SMZ) except for one patient in ICI group due to rapid death after admission. Most patients in both groups received corticosteroids use after diagnosis of PJP (ICI group vs non-ICI group: 90.1% vs 66.7%).

**Table 3:** PJP characteristics and treatment.

Variables	Non-ICI group (n=11)	ICI group (n=9)	p
**Onset time of PJP**	253.0 (185.1)	118.9 (60.9)	0.053
**Baseline Performance Status**	0.00 (0.0,1.0)	1.00 (0.0,1.75)	0.254
**CURB65**	1.00 (0,2.0)	1.0 (0.5,2.0)	0.754
**Bronchoscopic alveolar lavage**	11 (100%)	9 (100%)	1.00
**Diagnostic tools**			
Hexamine silver staining of BALF	4 (36.4%)	4 (44.4%)	1.00
PCR of BALF	4 (36.4%)	4 (44.4%)	1.00
mNGS of BALF	8 (72.7%)	6 (66.6%)	1.00
Blood test results			
CRP	43.6 (12.1, 102)	32.7 (19.28,64.35)	0.414
D-dimer	880 (540,1420)	1455 (655,2427.5)	0.305
Albumin	34.69 (4.60)	31 (5.00)	0.103
White blood cell count	5.77 (2.86)	6.05 (1.82)	0.801
Neutrophil count	4.74 (2.71)	4.79 (1.89)	0.962
Lymphocyte count	0.58 (0.48)	0.66 (0.21)	0.651
Eosinophil count	0.03 (0.01, 0.1)	0.04 (0.025, 0.075)	0.541
Hemoglobin	110.18 (21.87)	109.78 (24.94)	0.97
Platlets count	127.18 (63.74)	165.78 (38.91)	0.13
Cellular immunity			
Percentage of total T-cells	75.67% (10.46%)	71.87% (11.46%)	0.500
Total T-cells count	308.49 (110.32,842.80)	445.28 (280.80,608.31)	0.115
Helper T-cells (CD3+CD4+)count	138.75 (61.43,419.87)	278.08 (124.60,325.62)	0.203
Percentage of Helper T-cells (CD3+CD4+)	36.2% (8.48%)	41.53% (16.53%)	0.456
Cytotoxic T-cells (CD3+CD8+)count	157.68 (48.06,387.93)	175.20 (138.60,272.49)	0.643
Percentage of Cytotoxic T-cells (CD3+CD8+)	39.52% (6.28%)	28.78% (12.04%)	0.054
CD4/CD8 ratio	1.02 (0.64,1.29)	1.83 (0.90,1.88)	0.064
Fungal G test			
Bronchoalveolar lavage fluid	263.21 (167.98)	360.28 (240.78)	0.357
Blood	42 (10, 178)	38 (38, 38)	0.862
Corticosteroids use after diagnosis of PJP			
Use of corticosteroids	10 (90.1%)	6 (66.7%)	0.285
Cumulative dose of corticosteroids	3500 (1820, 4970)	960 (666, 3860)	0.115
Daily dose of corticosteroids	219 (193, 268)	179 (135, 238)	0.608
Duration of corticosteroids use	14 (10.25, 21)	9 (4, 16)	0.158
Other treatment			
TMP-SMZ	11 (100%)	8 (88.9%)	0.450
IVIG	1 (9.1%)	2 (22.2%)	0.566
Non-invasive ventilation	1 (9.1%)	1 (11.1%)	1.00
Invasive ventilation	1 (9.1%)	2 (22.2%)	0.566

All data are presented as No. (%), median (interquartile range), or mean (SD).

PJP, pneumocystis jiroveci pneumonia; CURB65, confusion, urea, respiratory rate, blood pressure and age; BALF, bronchoscopic alveolar lavage fluid; PCR, polymerase chain reaction; mNGS, metagenomic next-generation sequencing; TMP-SMZ, trimethoprim/sulfamethoxazole; CRP, C-reactive protein; IVIG, intravenous immunoglobins.

### Kaplan-Meier analysis

**Figure 2:**
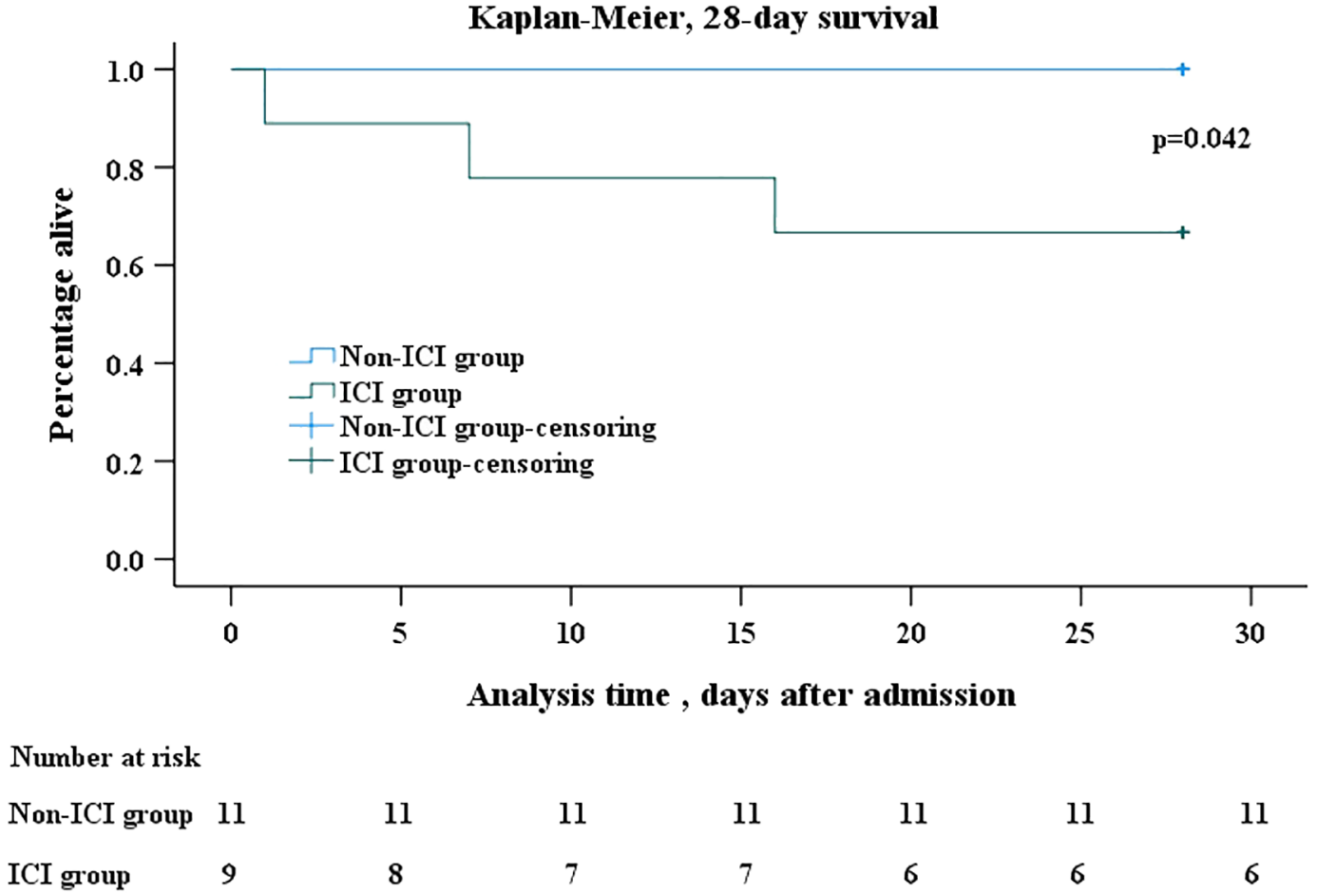
Kaplan-Meier survival analysis. Kaplan–Meier analysis of survival in 28 days after onset of PJP showed that mortality was significantly higher in ICI group than in non-ICI group (log rank, p=0.042). In the Kaplan–Meier analysis, censoring mean the total survival time for that subject cannot be accurately determined. The days after admission refer to the days after the patents’ admission to hospital due to PJP. The number at risk refer to patients infected with PJP who were still alive but at risk of death. PJP, *Pneumocystis jiroveci* pneumonia; ICI, immune checkpoint inhibitor.

Kaplan-Meier analysis revealed a significant difference in all-cause mortality after PJP onset between the two groups. Within 28 days after the onset of PJP, mortality was significantly higher in the ICI group than non-ICI group (33.3% vs 0, p=0.042) (**Figure 2**).

## Discussion

Our study reported that lung cancer patients with ICI use had a higher mortality rate after PJP infection than patients without ICI use. Our study also revealed that there was a trend towards shorter onset of PJP in patients receiving ICI. To the best of our knowledge, this study was the first retrospective study of the impact of ICI on mortality of PJP in lung cancer patients with including a comparator group. Although the overall incidence of PJP was low in patients with ICI use, it might bring severe consequence. So when there were patients presented with ground-glass opacity, physicians should be alert to the occurrence of PJP. In the future, prospective studies with larger sample size and a multi-center design are warranted to further verify the present results.

The full picture of PJP with ICI use remained mostly unknown. Most reported studies in this area were case reports/series (8). So far the most comprehensive study about PJP infection associated with ICI was an analysis base on the FDA FAERS database. The indications of ICI use in the study were lung cancer, melanoma, renal cell carcinoma and Hodgkin’s disease. In the study, 677 reports of PJP associated with ICI were identified, in which 300 (44.3%) PJP cases with fatal outcome (8). The ICI showed a lower signal of PJP than traditional chemotherapy. Male gender and age >65 years were predominant in PJP cases associated with across all ICI. With expanding use of ICI worldwide and continuing release of new ICI agents, the absolute number of PJP cases were expected to rise. More studies on the area were warranted, and the current study aimed to evaluate the mortality risk of ICI in PJP patients with lung cancer.

In current study, lung cancer patients with ICI use had a higher risk of death after PJP infection than patients without ICI use. As far as we knew, there was no similar report before. In the study conducted by Malek et al, researchers reported a similar infection-related mortality between patients treated with ICI combined with chemotherapy and those treated with chemotherapy alone. But those infectious episodes were most caused by bacteria, and none was PJP. So far it was generally believed that ICI use didn’t increase the risk of infection including PJP in cancer patients, but it remained unknown if ICI use increased the risk of death after PJP infection. Our study provided preliminary evidence to show ICI use might increase the risk of death after PJP infection. But our finding should be interpreted with caution, because of small sample size. So future multi-center studies with large sample size were needed to further verify our findings.

A possible reason why ICI use brought higher death risk was potential confounding checkpoint inhibitor-associated pneumonia (CIP) (18). As potentially fatal irAE caused by ICI, CIP was characterized by the presence of new infiltrative shadows on chest imaging and respiratory signs/symptoms related to a new emerging infiltration viewed on a chest imaging but excluding new infections or alternative etiologies (19). The incidence of CIP ranged from 2% to 38% in non-small cell lung carcinoma (NSCLC) in clinical trials and 4.8% to 39.3% in real-world studies (20).Although there was no consensus on the diagnostic evaluation of CIP, exclusion of new infection was a prerequisite for diagnosis (21). PJP and CIP may present with similar clinical manifestations. On chest Computer Tomography (CT), PJP presented as bilateral interstitial infiltrates and bilateral ground-glass exudate (22). But pulmonary ground-glass exudate, the classic radiographic pattern of PJP, was also a common radiographic pattern in CIP (23). By the current consensus definition, PJP and CIP couldn’t co-exist. But it was possible that patients had PJP and CIP at the same time, and the diagnosis of PJP based on detection of PJP from respiratory specimens excluded CIP. Consequently, the underdiagnosis of CIP may lead to improper management, resulting in increased mortality in patients. This may be a possible reason for the higher mortality rate in patients with ICI use. But with current definition of CIP, this possibility couldn’t be verified.

The mortality of PJP reported in current study was lower than previous reports. The current study reported a 28-day mortality of 33.3% in the ICI group and of 0 in non-ICI group. In published studies, the mortality of PJP in non-HIV-infected patients varied from 35% to 55% (5, 7, 24). A retrospective study conducted in Germany reported a mortality of 40% in patients with solid malignancies (5). This discrepancy might be explained by timely and accurate diagnosis of PJP via wide use of BALF sample and mNGS. Early diagnosis of PJP was critical for improving clinical outcomes, and early initiation of TMP/SMZ was significantly associated with reduced mortality (4, 17). But *in vitro* culture of *Pneumocystis jiroveci* was extremely difficult, and establishing a microbiological diagnosis of PJP remained a challenge. So the selection of the proper samples and detection methods was crucial in diagnosis of PJP. On one hand, the current gold standard sample for diagnosis of PJP was BALF, which was considered to be the highest quality respiratory sample (4). The main superiority of BALF was its proximity to the site of pulmonary infection, which was a good indication of the local lung environment (24). In the current study, BALF were collected from all patients and used for detection of PJ, which provided excellent sensitivity and specificity. On other hand, there were various detection methods of PJ, with different sensitivity and specificity. In the past, PJP was usually diagnosed based on direct-view techniques with different staining tests or immunofluorescence method, which had proven to be insensitive (25). Molecular tests such as PCR showed good sensitivity and specificity in diagnosis of PJP (25, 26). But suspicion of PJP was an essential prerequisite for physician to order PCR test, which were not necessarily the case in clinical practice. In recent years, mNGS had been developed to provide information on the Deoxyribonucleic Acid (DNA) sequence of microbial genomes (27). The mNGS allowed sequence-based identification of all potential pathogens, and it helped to identify specific pathogens for most unexpected cases, which might be lifesaving in critical scenarios. Previous reports showed that the mNGS was highly efficient in the diagnosing PJP (28–30). According to a meta-analysis, which included 418 cases diagnosed with PJP and 925 controls, the pooled sensitivity and specificity of BALF mNGS for diagnosis of PJP was 0.957 and 0.939 respectively (24). So the combination of BALF sample and mNGS might improve diagnosis efficiency of PJP, and timely and accurate diagnosis of PJP subsequently promoted targeted therapy against PJP and reduced mortality.

The profile of patients included in the study was in agreement with that of previous study. The ICI group had an average age of 69.11 ± 4.99 and 100% of male. In the FAERS database analysis of PJP, it was reported that male gender and age >65 years were predominant in PJP cases associated with ICI (8). This was also consistent with published case reports. By Xia’s account, on published case reports, 53.3% PJP cases associated with ICI were male and age more than 65 (8). This similarity lent more credibility to our findings.

The current study also revealed that there was a trend towards shorter onset of PJP in patients receiving ICI, although without statistical significance. As far as we knew, there was no similar report before. In the study by Malek, the results showed that duration between therapy initiation and infection onset was similar between patients treated with ICI combined with chemotherapy and those treated with chemotherapy alone (14). This finding suggested that ICI use might accelerate the onset of PJP in lung cancer patients, but it needed further validation.

The current study has a potentially important clinical implication for physicians. According to our findings, although ICI might not increase the incidence of PJP, it might cause higher mortality in PJP patients. It is well known that TMP-SMZ are very effective for both prevention and treatment of PJP (1, 31). So on one hand, the physicians should be in alert to determine those patients who are at greatest risk for developing PJP. Although so far no general strategy exists for identifying such populations, at least patients with long-term use of corticosteroids should be considered to be potential candidates for TMP-SMZ prophylaxis (2). On the other hand, when there is new onset of respiratory symptoms and ground-glass opacity on CT, physicians need to be vigilant regarding the possible development of PJP. In that case, mNGS for BALF samples should be preferred and used on time. The proper prophylaxis and timely treatment of PJP would bring significant survival benefit to the patients.

The major strength of our study was that it was the first to compare mortality in PJP patients with lung cancer between those treated with ICI and a concurrent control group treated without ICI. However, our study was subject to some limitations. First, the single-center retrospective design made it impossible to determine the causal relationship between ICI use and mortality. The retrospective design was also prone to missing data and bias due to reliance on documents available for review. Second, due to small size of PJP patients with lung cancer, no propensity score matching could not be applied to minimize bias. Third, despite the combined use of clinical symptoms, radiographic findings, and pathogen detection for PJP diagnosis, the possibility of including patients with PJ colonization cannot be fully eliminated.

## Conclusion

To the best of our knowledge, the present study provided preliminary evidence to show that lung cancer patients with ICI use had a higher mortality rate after PJP infection than patients without ICI use for the first time. Although the overall incidence of PJP was low in patients with ICI use, it might bring severe consequence. So when there were patients presented with ground-glass opacity, physicians should be alert to the occurrence of PJP. In the future, prospective studies with larger sample size and a multi-center design are warranted to further verify the present results.

## Data availability statement

Due to the potential compromise of patient privacy, the data sets generated and/or analyzed in the current study are not publicly available, but are available from the corresponding authors upon reasonable request. Requests to access these datasets should be directed to YM (email: 2314023@zju.edu.cn).

## Ethics statement

The studies involving humans were approved by Ethics Committee of Second Affiliated Hospital of Zhejiang University School of Medicine. The studies were conducted in accordance with the local legislation and institutional requirements. The ethics committee/institutional review board waived the requirement of written informed consent for participation from the participants or the participants’ legal guardians/next of kin because the non-interventional retrospective study was determined to be no greater than minimal risk.

## Author contributions

BF: Conceptualization, Software, Writing – original draft, Data curation, Formal analysis, Investigation, Visualization. XS: Conceptualization, Data curation, Writing – original draft, Investigation. WH: Conceptualization, Data curation, Writing – original draft, Formal analysis, Investigation. YZ: Conceptualization, Writing – original draft, Data curation. FC: Conceptualization, Data curation, Writing – original draft, Investigation. FL: Funding acquisition, Writing – review & editing, Methodology, Project administration. WL: Funding acquisition, Writing – review & editing, Methodology, Project administration. YM: Methodology, Writing – review & editing, Conceptualization, Project administration, Resources, Supervision, Funding acquisition, Validation.
